# Nutrition during pregnancy and early development (NuPED) in urban South Africa: a study protocol for a prospective cohort

**DOI:** 10.1186/s12884-018-1943-6

**Published:** 2018-07-24

**Authors:** Elizabeth A. Symington, Jeannine Baumgartner, Linda Malan, Lizelle Zandberg, Cristian Ricci, Cornelius M. Smuts

**Affiliations:** 1 Centre of Excellence for Nutrition, North-West University, Potchefstroom, South Africa; 2 Department of Life and Consumer Sciences, University of South Africa, Johannesburg, South Africa

**Keywords:** Maternal health, Maternal diet, Nutrition, Pregnancy, Birth outcomes, Early development, DOHaD

## Abstract

**Background:**

Adequate nutrition during pregnancy is important to ensure optimal birth outcomes, maternal health and offspring development. However, little is known about the dietary intake and nutritional status of pregnant women residing in urban South Africa. Therefore, the Nutrition during Pregnancy and Early Development (NuPED) cohort study was initiated to assess early nutrition-related exposures predictive of early childhood development in urban South Africa.

**Methods:**

The aims of this prospective cohort study are: 1) to assess dietary intake and nutritional status of urban pregnant women in Johannesburg, South Africa, and 2) to determine associations with birth outcomes, measures of maternal health, as well as measures of offspring health and development. Pregnant women (< 18 weeks’ gestation) (*n* = 250) are being recruited from primary healthcare clinics in Johannesburg and are followed-up at a provincial hospital. Participants’ dietary intake and nutrient status (focus on micronutrients and fatty acids) are assessed at < 18, 22 and 36 weeks’ gestation. Additional assessments during pregnancy include anthropometric and blood pressure measurements, obstetric ultrasound screens, and assessments of food security, maternal fatigue, prenatal depression, allergy, immune function, morbidity and gestational diabetes. At birth, maternal and neonatal health is assessed and an umbilical cord blood sample collected. Maternal and offspring health is followed-up at 6 weeks, as well as at 6, ≈7.5 and 12 months after birth. Follow-up assessments of mothers include anthropometric measures, diet history, nutrient status, blood pressure, breast milk composition, and measures of postnatal depression and fatigue. Follow-up assessments of the offspring include feeding practices, nutrient status, measures of growth, psychomotor, socio-emotional and immune development, morbidity, allergy, as well as analysis of the gut microbiome and the epigenome.

**Discussion:**

Ensuring adequate nutrition during pregnancy is one of the key actions endorsed by the South African Government to promote optimal early childhood development in an effort to eradicate poverty. The results from this study may serve as a basis for the development of context-specific nutritional interventions which can improve birth outcomes and long-term quality of life of the mother and her offspring.

## Background

Recent estimates indicate that 250 million children in low- and middle income countries are at risk of not reaching their developmental potential [1]. This is worrisome as suboptimal childhood development is associated with poorer adult health, well-being and productivity – leading to an intergenerational cycle of poverty. As the trajectories of physical and mental health later in life are determined fundamentally during the first 1000 days of life, both the World Health Assembly Nutrition Targets and the Sustainable Developmental Goals call for action to, among others, improve maternal, infant and young child nutrition in an effort to ensure sustainable social and economic progress.

Maternal health and nutrition gained heightened attention three decades ago with the publication of the developmental origins of health and disease (DOHaD) hypothesis. Barker and Osmond [2] proposed that the cardiovascular disease they observed in an adult population from England and Wales was at least partly associated with poor early nutrition, and specifically undernutrition in utero [3]. Undernourishment in utero can stress the foetus in ways that permanently affect physiological growth and development, and can be described as a reprogramming of the foetus’s developing phenotype [4]. Apart from the long-term health consequences, severe growth-restricted foetuses are at increased risk of stillbirth, and the live births have an increased risk of neonatal death, morbidity and permanent deficits in growth and neurocognitive development [5, 6].

Several maternal nutritional factors have been investigated in relation to adverse pregnancy outcomes, as well as offspring health and development [7–9]. The nutrients most studied during pregnancy include B-vitamins (particularly folic acid), vitamin D, iron, long-chain polyunsaturated fatty acids (particularly n-3 fatty acids) and iodine [8, 10]. However, adequate maternal intakes of zinc and vitamin A may also be important for optimal pregnancy outcomes, as well as for maternal and offspring health [11–13]. Furthermore, better overall diet quality has been associated with a lower risk for maternal perinatal depression and gestational weight gain, which in turn are risk factors for suboptimal offspring development [14–16].

The health of the adult South African population is a concern. South Africa has large economic disparities and 20% of the population are living in extreme poverty, indicating they cannot afford the minimum required food intake [17]. During a national survey in 2012, approximately 40% of the population were reported to have a monotonous diet based mainly on starches [18]. The country is undergoing a rapid nutrition transition characterised by changes in dietary patterns and nutrient intake alongside urbanisation [19, 20], which has resulted in a growing double-burden of under- and over-nutrition [20, 21]. Hence, not surprisingly, 31% and 13% of South African women of reproductive age are anaemic [22] and vitamin A deficient [18], respectively, while 68% of women are overweight or obese, and 46% hypertensive [22]. The effects are also seen in children, with 27% of under-fives being stunted [22]. Both stunting and poverty are known risk factors for poor child development [23]. Maternal short stature is, in turn, a risk factor for birth complications [24] – illustrating the intergenerational effect of poor nutrition.

Ensuring adequate nutrition during pregnancy is one of the key actions endorsed by the South African Government to promote optimal early childhood development in an effort to eradicate poverty [25]. The *Guidelines for maternity care in South Africa* [26] therefore recommend routine nutritional assessment – such as measuring mid-upper arm circumference and haemoglobin levels – and daily supplementation of 200 mg ferrous sulphate, 1000 mg calcium and 5 mg folic acid. However, studies show that the majority of South African women only seek or get access to public antenatal care in their second trimester of pregnancy [27–30], which might be too late for the routine supplementation programme or other interventions to be effective.

Very little is known about the diet and nutritional status of pregnant women in South Africa, specifically residing in urban areas. Furthermore, understanding the associations of maternal diet and nutritional status during pregnancy with birth outcomes, as well as offspring health and development in the South African population will form the basis for the development of context-specific nutrition interventions that may improve birth outcomes and long-term quality of life of the mother and her offspring. Consequently, the *Nutrition during Pregnancy and Early Development (NuPED)* cohort study was initiated to investigate nutritional status during pregnancy and assess early nutrition-related exposures predictive of early childhood development in urban South Africa.

### Aims of the study

**Fig. 1:**
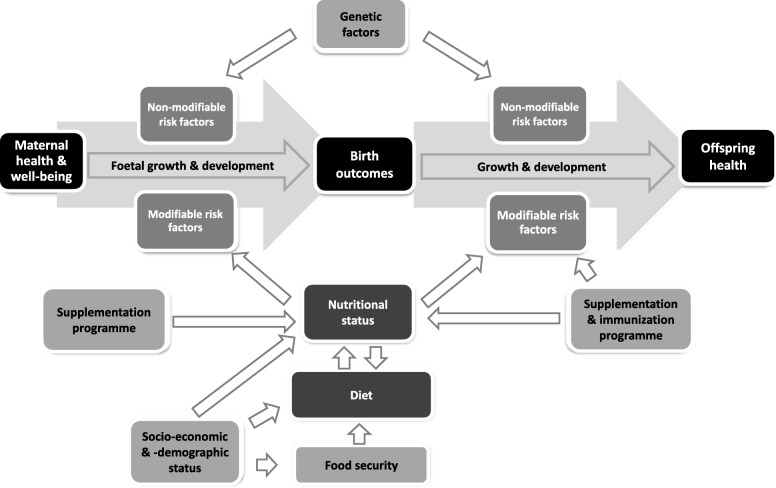
Simplistic conceptual framework of exposure and outcome variables in the NuPED study. The study investigates the indicated variables from early pregnancy to infants up to 12 months. The variables fall mainly in the modifiable risk factors category with the core interest being nutritional status. The outcome variables include birth outcomes such as birth weight and gestational age as well as postnatal growth and psychomotor development

The aims of the NuPED study are 1) to assess dietary intake and nutritional status of urban pregnant women in Johannesburg, South Africa, and 2) to determine associations with birth outcomes, measures of maternal health, as well as measures of offspring health and development. A simplistic conceptual framework showing the modifiable and non-modifiable exposure variables, as well as the outcome variables that will be determined to achieve the aims, is shown in Fig. 1.

## Methods/design

### Study design

This prospective cohort study follows 250 women throughout pregnancy to birth, and their infants up to 12 months of age. Briefly, data are collected early pregnancy, mid-pregnancy, late pregnancy and at birth. Postnatal assessments focus mainly on offspring health and development at 6 weeks, 6 months, 7.5 months (6 months + 6 weeks) and 12 months’ postnatal age.

Recruitment of participants started on 7 March 2016 and completion of data collection is expected in June 2019.

### Setting

The study is situated in Johannesburg, the largest city in South Africa. Recruitment of study participants takes place in two of the seven municipal regions of the city from which four primary health care clinics were identified. These clinics fall in the catchment area of Rahima Moosa Mother and Child Hospital (RMMCH). RMMCH is a provincial hospital focusing on maternal and paediatric healthcare, delivering more than 10,000 babies annually. Pregnancy data are collected at the antenatal care (ANC) clinic of RMMCH in addition to routine care. Birth data are collected in the relevant wards at RMMCH. Postnatal data are collected at the Empilweni Services and Research Unit (ESRU) at RMMCH. The execution of the study is coordinated by the Centre of Excellence for Nutrition of the North-West University.

### Study population

The study population is urban pregnant women attending ANC at either one of four selected primary health care clinics or at the ANC clinic of the hospital. Women interested to partake in the study are screened according to inclusion and exclusion criteria, and referred to RMMCH ANC clinic for signing informed consent and data collection if eligible.

The inclusion criteria applied during recruitment screening are: 1) Confirmed pregnancy and planning to deliver her baby at RMMCH; 2) < 18 weeks’ gestational age; 3) Born in South Africa, Lesotho, Swaziland, Zimbabwe, Botswana or Namibia and has been living in South Africa for at least 12 months; 4) Able to communicate effectively in one of the following languages: English, Afrikaans, Sotho, Zulu or Xhosa.

The exclusion criteria are 1) < 18 and > 39 years; 2) Multiple pregnancy; 3) Using illicit drugs (self-confessed); 4) Smoking (current and/or in past year); 5) Known non-communicable disease (NCD) namely diabetes, renal disease, high cholesterol, and hypertension; 6) Known infectious disease namely tuberculosis and hepatitis; 7) Known serious illness namely cancer, lupus or psychosis.

Even though women with infectious disease are excluded, women who are HIV positive are still included. Due to the high prevalence of HIV in the country (36% of women aged 30–34 years [31]), their inclusion will make generalisation to the wider South African population a possibility.

### Recruitment and consent procedures

Consecutive sampling is applied, thus, all accessible pregnant women at the recruitment sites may form part of the sample, if they meet the inclusion and exclusion criteria, arrive at study site on their booked date and sign informed consent. All pregnant women in the waiting areas of the ANC clinics are informed about the study. Those interested, receive a study information leaflet and are screened for eligibility individually in a private space upon which a booking date is supplied if eligible. The informed consent form is given to the participant to read, consider and discuss with her partner and/or family. Upon arrival at RMMCH on the booked date, the trained fieldworkers explain the informed consent form in local languages and all are given the opportunity to ask questions. All are assured that participation is voluntary and that participation or non-participation in the study will not affect their clinical care. All research participants provide written informed consent before data collection. Written informed consent is again obtained before infant assessments at 6 weeks postnatally.

### Data collection

**Fig. 2:**
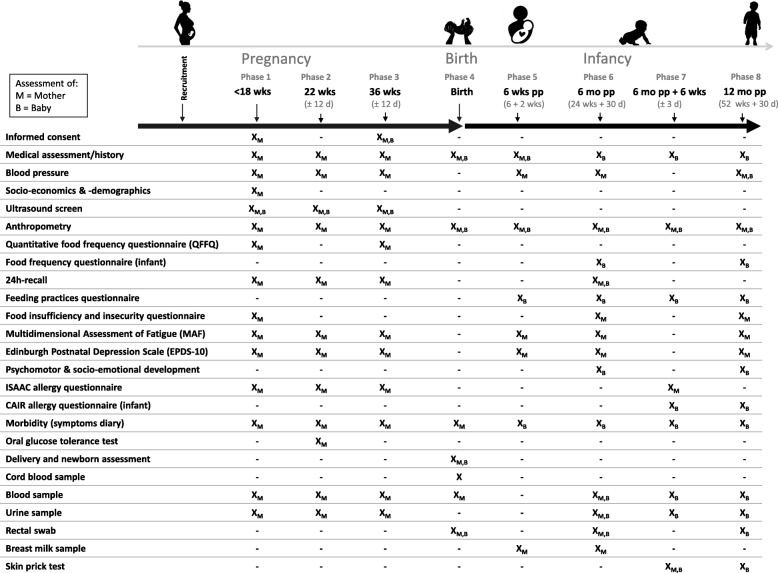
Data collection per phase during the NuPED study. Prenatal data collection time points are at < 18 weeks’ gestation (phase 1); ±22 weeks’ gestation (phase 2); ±36 weeks’ gestation (phase 3) and at birth (phase 4). Postnatal data collection time points are at infant postnatal age of 6 weeks (phase 5); 24 weeks (phase 6); 6 weeks post measles immunisation (± 3 days) (phase 7) *or* 30 weeks (+ 30 days) if measles immunization was not given between 24 and 28 weeks; and 52 weeks (+ 30 days) (phase 8). wks, weeks; d, days, pp, postpartum

Figure 2 summarises the measurements and time points of data collection throughout the project. There are eight data collection time points (here forth referred to as phases). All data are collected either by health professionals on site or by trained fieldworkers. Phase 1 data are collected at < 18 weeks’ gestation (as confirmed by obstetric ultrasound). These data will supply information on the nutritional status of women early in their pregnancy. It is important to note that in Johannesburg only 45% of women access ANC before 20 weeks gestation and only 23% in their first trimester (as reported from other urban areas) [30]. Thus, for practical purposes, the *early pregnancy window* was set at < 18 weeks’ gestation. Phase 2 data are collected at 22 weeks’ gestation (window ±12 days) when anomaly ultrasounds are typically scheduled. Phase 3 data are collected at 36 weeks’ gestation (window ±12 days). Study midwifes and/or fieldworkers collect birth data (phase 4) within a window of 12 h. Postnatal data are collected at 6 weeks (+ 14 days) (phase 5); 24 weeks (+ 30 days) (phase 6); 6 weeks post measles immunisation (± 3 days) (phase 7) *or* 30 weeks (+ 30 days) if measles immunization was not given between 24 and 28 weeks; and 52 weeks (+ 30 days) (phase 8) postnatal age. The purpose of the phase 7 data collection point specifically is to assess measles immunoglobulin G (IgG) as marker of response to immunisation at 6 months and immune function.

#### Maternal socio-economics and -demographics

Socio-economic and -demographic data are collected at phase 1 by means of a structured interview. Data include date and country of birth, marital status, educational level, home language, employment status, household income, number of members in the household, and beneficiaries of social grants. Lastly, living standards data are obtained to allow classification according to the Living Standards Measure (LSM) developed by the South African Audience Reference Foundation (SAARF) [32]. This measure is widely used in South Africa to describe the socio-economic status of the population [33].

#### Maternal household food insufficiency and insecurity

The level of the participating women’s household food insufficiency and insecurity is assessed at phase 1 in pregnancy and again at phases 6 and 8 postnatally. In a structured interview, women are asked questions on food insecurity and child hunger using the Community Childhood Hunger Identification Project (CCHIP) index [34] that was also used to determine the status of food security in previous national surveys in South Africa [35]. Furthermore, women were asked a validated, single-item question on food insufficiency –“*How many days in the past week have you gone hungry? By this I mean days when you felt you didn’t have enough to eat*.”–that was previously used to determine food insufficiency in pregnant women in South Africa [36].

#### Maternal dietary intake

Maternal dietary intake data are obtained by means of two dietary assessment methods, namely the 24-h recall (24-HR) and a quantified food frequency questionnaire (QFFQ). Both methods are interviewer administered by using standardised probing questions [37]. Standard measuring equipment, common size containers (e.g. cups, bowls and glasses) as well as two- and three-dimensional food models are being used to assist in quantifying portion sizes.

A single 24-HR, which obtains details about nutritional supplement use as well, is administered at phases 1, 2 and 3 in pregnancy, as well as at 6 months postnatally (phase 6). Each participant is requested to recall all foods and drinks consumed the previous day from when she woke up until the next day the same time. The recall is done chronologically unless the participant wishes to recall randomly. The purpose of the single 24-HR is to describe the average intake of the group [38]. All supplement use, as well as food cravings and aversions, are additionally recorded daily by participants on a calendar.

The second dietary assessment method, the QFFQ, is completed at phases 1 and 3. It was validated for the population in the Transition and Health during Urbanisation of South Africans (THUSA) study [39] and its reproducibility was proven [40, 41]. It was also used previously to assess individual and total omega-3 and omega-6 fatty acid intake in a rural and urban South-African population [42]. This QFFQ includes a list of typically consumed foods and minor changes were made to the questionnaire according to vernacular used by the study population in that particular area. Participants are asked according to the ~140 food items listed in the QFFQ, the type/brand, cooking methods, frequency and the amount of all food and drink consumed in the past 4 weeks.

For the QFFQ data, portion sizes are converted to grams per week per food item, by two registered dietitians/nutritionists. For the 24-HR all portion sizes are converted to grams per day per food item. The resources to assist with this include the Condensed Food Composition Tables for South Africa [43] and the South African Medical Research Council (SAMRC) Food Quantities Manual [44].

#### Maternal anthropometric measurements

Maternal weight and mid-upperarm circumference (MUAC) are obtained at each phase (1 to 8), and height only at phase 1 and 5. All measurements are done twice and recorded to the nearest 0.05 kg for weight, 0.1 cm for MUAC and height. Standardised methods of the International Society for the Advancement of Kinanthropometry [45] are used with a calibrated digital scale for weight, a mobile stadiometer for height; and non-stretchable metal measuring tape for MUAC.

#### Maternal medical assessment and history

Medical history is obtained at each prenatal visit (phases 1 to 3) by means of participant responses and inspection of medical files. Information includes medication use (including vaccines), HIV status, obstetric history, hospital admission during pregnancy, use of alcohol, and exposure to passive smoking. At the first postnatal visit (phase 6) a follow-up is made on the maternal medical history at birth. Blood pressure is measured at each prenatal visit (phases 1 to 3) as well as postnatally (phases 5, 6 and 8) according to international guidelines [46] using calibrated equipment. Appropriate sized cuffs are used for obese participants.

Standard procedures are used for a 2-h 75 g oral glucose tolerance test (OGTT) between 24 and 28 weeks gestation to determine development of gestational diabetes mellitus [47].

#### Maternal morbidity

Maternal morbidity symptoms are assessed from enrolment to birth using a daily calendar. Mothers are instructed on how to complete the calendar and to return completed calendars at each visit. The infectious morbidity symptoms assessed are fever, headache, diarrhoea, nasal discharge and coughing. Other possible pregnancy-related symptoms included are constipation, nausea, vomiting, extreme tiredness and heartburn. Any medication and supplementation use is also recorded daily.

#### Maternal allergy assessment

The International Study for Asthma and Allergies in Childhood (ISAAC) questionnaire [48] is used to assess allergy symptoms in maternal participants at phases 1 to 3 during pregnancy, and 7 postnatally. Additionally, skin prick tests for common allergens are used at phase 7 postnatally to assess sensitisation [49]. The questionnaire is designed to assess rhinitis, asthma and eczema in children and has been used successfully in an older black population in South Africa [50]. A positive score on any of these three symptoms indicates an allergic phenotype.

Maternal skin prick tests are performed by a medical doctor according to the procedure described in the Allergy Society of South Africa’s position statement on skin prick testing [49]. In mothers, sensitisation to a house dust mite mixture including Dermatophagoides farinae, as well as German cockroach, mould mixture, cat and dog dander, maize pollen, Bermuda grass and *Quercus robur* (English oak), Eucalyptus, Cypressus arizonica (Arizona cypress), *Platanus hybrida* (London plane) and Acacia trees are measured. A diagnosis of 1) sensitised with clinical symptoms, 2) sensitised and clinically tolerant, 3) sensitised and unknown clinical reactivity, 4) not sensitised with clinical symptoms, 5) unknown sensitization with clinical symptoms or 6) not sensitized with no clinical symptoms is made. The mother is given medical advice and referred if necessary.

#### Maternal depression and fatigue

Perinatal depression is assessed at phases 1 to 3 during pregnancy and at phases 5, 6 and 8 postnatally using the Edinburgh Postnatal Depression Scale (EPDS). The EPDS is a 10-item scale assessing depressive symptoms experienced in the past 7 days [51], which has been validated for assessing perinatal depression in African settings, including South Africa [52]. Maternal fatigue is assessed at the same time points using the Multidimensional Assessment of Fatigue (MAF) scale, which was shown to be a reliable and valid measure of fatigue in pregnant and postpartum women [53]. Both questionnaires are interviewer administered.

#### Foetal ultrasonography: Gestational age and foetal growth

Foetal ultrasonography examination is carried out by an obstetrician at the first data collection time point to confirm gestation. Estimation of foetal crown rump length and/or biparietal diameter or femur length between 6 and 18 weeks’ gestation indicates an accuracy within 5–7 days [54]. Foetal crown-rump length is used to determine gestational age of participants in their first trimester [55]. For participants in their second trimester, a combination of multiple biometric parameters (biparietal diameter, head circumference, abdominal circumference, and femur length) are used to determine gestational age [55]. Ultrasound is also used to determine the number of foetuses and confirm foetal movement, as well as foetal growth at 22 and 36 weeks’ gestation (phases 2 and 3).

#### Birth and neonatal assessments

Maternal data collected at birth (phase 4) are obtained from maternal medical files and include hospital admission and discharge dates and times, mode of delivery, induction/augmentation of delivery, type of anaesthetic or pain relief if any, rapid plasma reagin (RPR) status (indicative of syphilis infection), HIV status, rhesus negative status and presence of maternal diabetes mellitus. If the delivery is induced or caesarean section conducted, the reason for this intervention is obtained. The study nurses obtain maternal weight before birth with a calibrated digital scale.

Neonatal data collected at birth from the medical file include date and time of birth, gender, Apgar score (at 1 and 5 min) [56], vital signs, medical interventions required, foetal distress and presence of meconium stained liquor. Four identically trained study nurses obtain newborn anthropometry (weight, midarm circumference (MAC), crown-heel length (CHL), head circumference (HC) and thoracic circumference (TC)) within 12 h of birth [57]. If the measurements cannot be taken by the study nurses, hospital records are used to obtain anthropometrical data (using the same calibrated infant scale).

Newborn weight is measured with a calibrated digital infant scale to the nearest 10 g. In order to minimize intra-observer variability all circumferences and CHL are measured with an inelastic tape to the nearest 0.5 cm (metal measuring tape not used to prevent possible lacerations). CHL is measured by placing the newborn in supine position on the tape measure on a flat surface with all limbs extended and measurement taken from vertex to heel of foot, with foot held in a perpendicular position to the leg.

#### Infant dietary intake and feeding practices

Data on infant feeding practices are collected at each postnatal phase (5 to 8). Mothers are asked how soon after birth the infant was breastfed, if the infant is currently being breastfed and if not, the duration of breastfeeding. All mothers are asked details about any other food or drink (including infant formula, medicine and supplements) given to the infant.

An unquantified food frequency questionnaire for the infant is administered at phases 6 and 8 for qualitative assessment (types and frequency) of milk and complementary feeding at 6 and 12 months postnatally. An adapted questionnaire previously used in the South African context is used [58]. Frequency of the type of food eaten during the past month can be reported by the mother as *every day*; *most days* (not every day, but at least 4 times per week); *once a week* (less than 4 times per week, but at least once per week) or *never*.

A single 24-HR for the infant is administered at phase 6 for quantitative assessment (macro- and micronutrient intakes) of intakes at 6 months postnatally. Similar methods and aids are used as described for maternal dietary intake. However, smaller bowls and different sizes of small spoons are used for infant dietary intake to ease realistic reporting for the mother. Also, emphasis is placed on dished food not eaten and the amount left in the bowl to ensure actual intake is reported.

#### Infant anthropometric measurements

Infant growth is assessed at each postnatal visit. Before measurements are taken, the infant is assessed for presence of oedema. The infant is weighed on a calibrated scale with minimum of clothing, namely only a vest, and without a nappy; and recorded to the nearest 5 g.

Recumbent length of the infants is taken by means of an infantometer to the nearest 0.1 cm. All foot and headwear is removed before measurements are taken. The measurement is taken with the infant lying on his/her back on the infantometer, legs extended with the head and foot board making contact with the infant.

#### Infant medical assessments

A medical doctor performs a general and physical medical assessment of infants at each postnatal visit. The infant assessment includes HIV status history and a general, ear, nose and throat, respiratory, cardiovascular, abdominal and neurological examination as well as any current complaints. It also includes a medical plan for the infant.

#### Infant morbidity

Infant morbidity assessment is performed at each postnatal visit by a medical doctor with a structured questionnaire. A morbidity calendar, kept daily by the mother/caregiver, is used as reference. The calendar and symptoms are explained to the mother at birth and each postnatal visit up to phase 7, whereby each new section of the calendar is handed to the mother for return at the next visit. Symptoms assessed are fever, diarrhoea, vomiting, nasal discharge, coughing, diaper and other rash. Any unscheduled visits to a medical facility and medicine given to the infant are also recorded. The medical doctor diagnoses and determines the duration of each morbidity event using the structured questionnaire with reference to the morbidity calendar.

#### Infant allergy assessment

A medical doctor assesses the allergy phenotype and sensitisation of infants with the Childhood Allergy and Immunology Research (CAIR) questionnaire and skin prick tests at phases 7 and 8 postnatally. The CAIR questionnaire was developed by the School of Paediatrics and Child Health of the University of Western Australia and is designed to assess asthma, rhinitis and eczema in infants.

Infant skin prick tests are performed at phases 7 and 8 by a medical doctor according to the procedure described in the Allergy Society of South Africa’s position statement on skin prick testing [49]. Skin prick tests to determine sensitisation to common allergens are common medical practice in infants older than 4 months [59, 60]. In infants, sensitization to a house dust mite mixture including Dermatophagoides farinae, German cockroach, mould mixture, cat and dog dander, maize pollen, Bermuda grass, chicken egg, cow’s milk, cod fish, peanuts, wheat and soy bean flour and potato are measured. A diagnosis of 1) sensitised with clinical symptoms, 2) sensitised and clinically tolerant, 3) sensitised and unknown clinical reactivity, 4) not sensitised with clinical symptoms, 5) unknown sensitization with clinical symptoms or 6) not sensitized with no clinical symptoms is made. The infant’s medical plan is managed accordingly.

#### Infant immune response

The infant’s IgG response to measles immunisation is assessed at phase 7, which is 6 weeks after measles immunisation was administered at the study site. The measles immunisation in South Africa forms part of the National Expanded Programme for Immunisation and permission to administer it at the study site has been granted by the Department of Health of Gauteng Province and the City of Johannesburg. Response to an immunisation is regarded as a good marker to measure immune function in vivo [61] and the response will be in the log phase 6 weeks after immunisation, which is the most sensitive stage to measure differences in response among infants.

#### Infant psychomotor and socio-emotional development

Psychomotor and socio-emotional development of the offspring is being assessed using the Protocol for Child Monitoring –Infant version (PCM-I), which combines both parental report and direct observation by trained assessors to provide a comprehensive evaluation of a child’s motor skills, cognition, language, personal and socio-emotional development [62]. The PCM-I consists of items derived from: 1) the Kilifi Developmental Inventory (KDI) [63], previously used by the investigators to determine psychomotor development in an infant population in South Africa [64], 2) the Developmental Milestone Checklist (DMC-II) [65, 66], and 3) the Profile of Social-Emotional Development (PSED), which is based in part on the Brief Infant/Toddler Social Emotional Assessment [67].

#### Biological sample collection

Venous blood (42 ml) is drawn from the participating women into labelled EDTA-coated, serum and trace element free evacuated tubes at phases 1–4 during pregnancy and at phase 6 postnatally. At birth (phase 4), umbilical cord blood samples are taken immediately after the separation of the newborn from the umbilical cord and before placental delivery into labelled EDTA-coated, serum and trace element free evacuated tubes. Venous blood from the infant (3 ml) is drawn at phases 6, 7 and 8. Dry blood spots are collected on filter paper cards (Whatman, Inc) immediately after blood collection (maternal, cord and infant). The filter paper cards are allowed to dry at room temperature for 24 h, placed in ziplock bags with desiccants, and stored at − 20 °C until analysis. In case venous blood cannot be obtained from the infants, capillary blood is being collected by foot venepuncture.

Venous blood is processed within 1 h after blood draw; plasma/serum separated and red blood cells washed twice with normal saline. Buffy coats are stored in 1:1 vol: vol RNA later (Ambion).

Midstream spot urine samples (5 ml) are collected from the participating women at phases 1, 2 and 3 during pregnancy and at phase 6 postnatally into a urine collection cup. From the infants, a 2–5 ml urine sample is collected at phases 6, 7 and 8 using adhesive paediatric urine collection bags. Urine samples are transferred into labelled microtubes and stored at − 20 °C within 4 h.

Breast milk samples (fore-milk) are collected from lactating mothers at phases 5 and 6 as described previously [68].

Rectal swabs (FLOQSwab, COPAN) are collected from both the mother and the baby at phases 4 and 6 of data collection. The mucosal microbe sample is taken approximately ±3 cm into the anal canal, beyond the anal verge. After collection the cotton bud end of the swab with the collected sample is immediately preserved in RNAlater (Ambion) and stored at − 20 °C.

Biological samples are processed on site and stored at − 20 °C for a maximum of 14 days. Thereafter, frozen samples are transported to the North-West University for storage at − 80 °C until analysis. Storage temperature is monitored and logged for the entire duration of the study.

#### Biochemical analyses

Haemoglobin is determined on site in whole blood (20 μL) using HemoCue (Hb 201+, Ängelholm, Sweden). The iron status indices, ferritin and transferrin receptor, as well as the vitamin A status indicator retinol binding protein will be determined using the Q-Plex™ Human Micronutrient Array (7-plex, Quansys Bioscience, Logan, UT, USA) [69]. This multiplex immune-assay also includes the acute phase proteins C-reactive protein (CRP) and alpha1-acid glycoprotein (AGP), as well as the malaria marker HRP2 and thyroglobulin, which is a marker of iodine status. Urinary iodine concentrations are determined in spot urine samples using a modification of the Sandell-Kolthoff reaction with spectrophotometric detection [70]. Vitamin A and E status will be determined using high pressure liquid chromatography (HPLC) and ultraviolet light detection [71]. Vitamin D status will be determined by measuring total 25-Hydroxyvitamin D [25(OH)D] concentrations in serum using liquid chromatography tandem mass spectrometry (LCMSMS) [72]. Fatty acids in red blood cell total phospholipids are determined using gas chromatography tandem mass spectrometry (GCMSMS) [73]. Zinc concentrations are determined in serum using atomic absorption spectrometry [74].

Thyroid hormones (thyroid stimulating hormone, thyroglobulin, total thyroxine) will be determined in whole blood spots by using electrochemiluminescence immunoassays. Lipid-derived immune modulators will be determined with LCMSMS [75]. Cytokines and hepcidin will be determined using ELISA.

Kynurenine pathway metabolites (mediates interactions between immunological and neurological function) will be determined using LCMSMS [76]. Brain-derived neurotrophic factor (BDNF) as a potential marker of neuronal growth and differentiation will be determined using ELISA [77].

Gut microbiome profiling will be done by isolating microbial DNA from the collected rectal swabs using Qiagen Stool minikit and analysing the 16S rRNA genes DNA sequences on the Ion Torrent 16S metagenomics solution offered by ThermoFisher Scientific.

Targeted epigenetic marks, specifically DNA methylation signatures, will also be assessed in the context of the primary and secondary outcomes of this study. Gene specific DNA methylation will be assessed using the Qiagen EpiTech system. Both, the EpiTect Methyl II Signature and EpiTect Methyl II Complete PCR Arrays (Qiagen) will be considered.

Targeted genotyping of genes of interest in the context of fatty acid, lipid and micronutrient metabolism will also be investigated following the Ion AmpliSeq Targeted Sequencing approach using the Ion *Chef*™ and Ion *S5*™ Systems (ThermoFisher).

### Data management and analysis

#### Sample size calculation

The number of participants to sample has been calculated using the G*Power 3.1.9.2 statistical programme [78]. The statistical calculation involved is the linear multiple regression: fixed model, single regression coefficient. The calculation was based on a small effect size F^2^ of 0.05; probability of error (alpha) of 5%; a power of 80% and ten predictors on the birth outcome “low birth weight”. The result was that 196 participants will be required. Taking into consideration that participants may opt out of the project (at 25% rate), it is calculated that a minimum of 245 participants should be recruited. We intended to recruit a minimum of 250 participants. However, should the researchers be able to obtain additional funding, additional participants may be included.

#### Data management

Data are managed by two dedicated data managers. Raw data are captured and saved in password protected Microsoft Access documents with passwords known only to the operator responsible for data imputing and the principal investigators. A second person checks 20% of all the captured data randomly and notes and corrects any errors. If there are more than 5% errors, respective data are re-captured. The final version of the database will be stored under protected zipped files. Data are collected on dual core electronic archives with automatic backup. Information of the single datasets are stored using anonymous IDs. The document linking anonymous IDs to participants will be collected and stored separately.

Dietary data are captured in Microsoft Excel (Microsoft Corporation, Washington, USA) and all electronic entries are double checked by a registered dietitian for the correct food code and a reasonable amount reported. Analyses will be done by the SAMRC by linking data to the most recent food composition database. Data will then be screened by means of range checks and for outliers in total energy, protein, fat, vitamin A and vitamin C intake.

#### Data analysis

Overall, data processing and statistical analysis are performed using the SAS statistical package (SAS, Cary, NC, USA). Analysis of baseline (phase 1) data will be conducted to describe the nutritional status and basic socio-economic characteristics of the pregnant women. Data will be tested for outliers and normality by means of Q-Q plots and histogram visual inspection. Test for normality will be performed by the Shapiro-Wilk test. Normally distributed data will be expressed as means ± SD; non-normally distributed data will be expressed as medians (25th percentile, 75th percentile).

Data will be analysed cross-sectionally to determine associations between variables at each time point by using appropriate statistical methods (e.g. multiple linear regression analysis, ANCOVA, logistic regression analysis), adjusting for potential covariates.

Data will be analysed prospectively to determine associations between variables at different time points (longitudinally) by using appropriate statistical methods (e.g. linear mixed effects models), adjusting for potential time-dependent and static covariates.

Data will also be analysed retrospectively in matched-control sub-studies by determining associations between observed outcomes and variables collected at previous time points.

The level of significance will be set at *P* < 0.05.

## Discussion

The importance of perinatal nutrition and its role in offspring health, is recognised [79]. Nutrition during pregnancy is an important factor associated with both maternal and infant health outcomes [80]. To date, however, South African public health nutrition interventions for pregnant women are limited to folic-iron and calcium supplementation, while it is highly likely that the diet of pregnant women living in South Africa is lacking vital micronutrients and essential fatty acids beyond those supplied, or is even containing excessive amounts of specific micro- and macro-nutrients. In order to advocate evidence-based healthcare policy and practice, the identification of nutrient deficiencies and poor eating patterns of pregnant women in South Africa, which are associated with adverse neonatal outcomes and delayed early offspring development, is imperative.

Little is known about the dietary behaviour and nutritional status of pregnant women living in South Africa. To the best of our knowledge, this is the first South African study focusing on the assessment of both maternal dietary intake and nutritional status in women pre- and postnatally and to investigate associations with outcomes of maternal and infant health. This study is novel due to the comprehensive set of nutrition related data and indicators of maternal and infant health being obtained in a South African setting. Therefore, this explorative project will contribute to identifying factors that may be targeted in future pre- and/or post-conception maternal interventions for optimal offspring development and possibly reduction in adult NCD risk.

This study also has its challenges. The inclusion and exclusion criteria for participants were designed as such to obtain a sample of generally healthy women, who are able to speak the local language and who could be followed-up from early pregnancy until the infants are 12 months old. South African statistics show that only 52% of women attend antenatal care before 20 weeks’ gestation [30], thus access to women early in pregnancy is restricted and limits enrolment into the study. Furthermore, many healthcare facilities in Johannesburg serve a predominantly migrant population [81] posing a challenge for longitudinal data collection. Additionally, these women may choose to eat traditional foods [82] that do not form part of the South African Food Database for nutritional analysis; and may not be able to speak a local language [83] hampering detailed reporting during dietary assessments. Thus, migrating women or those unable to speak local languages could not be included in the study.

A limitation of the study is that women who fit the inclusion criteria are invited to join the study by visiting the data collection site at an agreed date. Having this option of attending may contribute to self-selection bias. Our recruitment data to date indicate that of those invited to take part in the study; only approximately 50% arrive at the data collection site on the booked date.

Even so, this study will provide a comprehensive and unique database from an urban South African setting which will allow for cross-sectional, prospective and retrospective analyses to describe the nutritional status and dietary intake of pregnant women, and to determine associations with health outcome measures. These results will supply context to intervention studies with the aim to improve maternal as well as offspring health in South Africa.

## Abbreviations

24-HR: 24-h recall
AGP: Alpha1-acid glycoprotein
ANC: Antenatal care
BDNF: Brain-derived neurotrophic factor
CHL: Crown-heel length
CRP: C-reactive protein
DOHaD: Developmental Origins of Health and Disease
ELISA: Enzyme-linked immunosorbent assay
ESRU: Empilweni Services and Research Unit
GC-MS/MS: Gas chromatography-mass spectrometry
HC: Head circumference
HPLC: High-performance liquid chromatography
HRP2: Histidine-rich protein 2
MAC: Mid-arm circumference
MUAC: Mid-upper arm circumference
NCD: Non-communicable disease
NuPED study: Nutrition during pregnancy and early development study
OGTT: Oral glucose tolerance test
QFFQ: Quantified food frequency questionnaire
RMMCH: Rahima Moosa Mother and Child Hospital
RPR: Rapid plasma reagin
SD: Standard deviation
TC: Thoracic circumference
